# Supplementary material for: Modeling glioblastoma heterogeneity as a dynamic network of cell states
Source: Mol Syst Biol. 2021 Sep 16;17(9):e10105. doi: 10.15252/msb.202010105 (PMC8444284; doi:10.15252/msb.202010105)
Supplement: Supplementary file 6 — Source Data for Figure 5 [file MSB-17-e10105-s004.zip › Figure5A_sourcedata/GSEA_3071/nosigdb_stateVI.GseaPreranked.1622795169056/DOROSZKO_U3180MG_CLUSTER_4.html]

Details for gene set DOROSZKO\_U3180MG\_CLUSTER\_4[GSEA]

|  || Dataset | state63071 |
| Phenotype | NoPhenotypeAvailable |
| Upregulated in class | na\_pos |
| GeneSet | DOROSZKO\_U3180MG\_CLUSTER\_4 |
| Enrichment Score (ES) | 0.21584184 |
| Normalized Enrichment Score (NES) | 1.493551 |
| Nominal p-value | 0.03521127 |
| FDR q-value | 0.07664244 |
| FWER p-Value | 0.67 |
Table: GSEA Results Summary

  

Fig 1: Enrichment plot: DOROSZKO\_U3180MG\_CLUSTER\_4      
 Profile of the Running ES Score & Positions of GeneSet Members on the Rank Ordered List

  

| PROBE | GENE SYMBOL | GENE\_TITLE | RANK IN GENE LIST | RANK METRIC SCORE | RUNNING ES | CORE ENRICHMENT || 1 | CDC20 |  |  | 15 | 1.073 | 0.0275 | Yes |
| 2 | SNAPC1 |  |  | 96 | 0.738 | 0.0268 | Yes |
| 3 | ECI2 |  |  | 105 | 0.735 | 0.0463 | Yes |
| 4 | EZR |  |  | 148 | 0.667 | 0.0542 | Yes |
| 5 | CCNB1 |  |  | 181 | 0.620 | 0.0635 | Yes |
| 6 | LDHA |  |  | 197 | 0.604 | 0.0772 | Yes |
| 7 | RUVBL2 |  |  | 212 | 0.595 | 0.0908 | Yes |
| 8 | TIMM9 |  |  | 224 | 0.586 | 0.1050 | Yes |
| 9 | LGALS1 |  |  | 323 | 0.526 | 0.0931 | Yes |
| 10 | PTTG1 |  |  | 344 | 0.514 | 0.1027 | Yes |
| 11 | PRR13 |  |  | 345 | 0.514 | 0.1178 | Yes |
| 12 | PRMT5 |  |  | 372 | 0.502 | 0.1254 | Yes |
| 13 | CCT7 |  |  | 460 | 0.467 | 0.1148 | Yes |
| 14 | CCT8 |  |  | 474 | 0.463 | 0.1248 | Yes |
| 15 | TROAP |  |  | 479 | 0.461 | 0.1373 | Yes |
| 16 | IGFBP4 |  |  | 571 | 0.433 | 0.1245 | Yes |
| 17 | AURKA |  |  | 572 | 0.432 | 0.1373 | Yes |
| 18 | RBM42 |  |  | 577 | 0.431 | 0.1489 | Yes |
| 19 | RANGRF |  |  | 605 | 0.423 | 0.1538 | Yes |
| 20 | HMMR |  |  | 681 | 0.404 | 0.1447 | Yes |
| 21 | MRPL47 |  |  | 687 | 0.402 | 0.1552 | Yes |
| 22 | PLIN3 |  |  | 781 | 0.380 | 0.1403 | Yes |
| 23 | CIB1 |  |  | 807 | 0.375 | 0.1444 | Yes |
| 24 | ACTN1 |  |  | 845 | 0.369 | 0.1449 | Yes |
| 25 | TXNDC17 |  |  | 865 | 0.366 | 0.1504 | Yes |
| 26 | SPA17 |  |  | 914 | 0.358 | 0.1475 | Yes |
| 27 | NUP37 |  |  | 928 | 0.356 | 0.1544 | Yes |
| 28 | KIF20A |  |  | 961 | 0.351 | 0.1558 | Yes |
| 29 | PSME2 |  |  | 962 | 0.351 | 0.1662 | Yes |
| 30 | JPT1 |  |  | 965 | 0.350 | 0.1759 | Yes |
| 31 | CCNB2 |  |  | 967 | 0.350 | 0.1860 | Yes |
| 32 | PRR11 |  |  | 969 | 0.350 | 0.1960 | Yes |
| 33 | AIMP2 |  |  | 973 | 0.349 | 0.2055 | Yes |
| 34 | UBE2S |  |  | 974 | 0.349 | 0.2158 | Yes |
| 35 | NUDT11 |  |  | 1028 | 0.340 | 0.2110 | No |
| 36 | EIF4G2 |  |  | 1100 | 0.330 | 0.2009 | No |
| 37 | SORD |  |  | 1239 | 0.311 | 0.1714 | No |
| 38 | COPRS |  |  | 1274 | 0.306 | 0.1709 | No |
| 39 | EMP3 |  |  | 1278 | 0.306 | 0.1791 | No |
| 40 | TMSB10 |  |  | 1289 | 0.305 | 0.1853 | No |
| 41 | NEK2 |  |  | 1307 | 0.302 | 0.1894 | No |
| 42 | STIP1 |  |  | 1311 | 0.302 | 0.1975 | No |
| 43 | MORF4L2 |  |  | 1367 | 0.295 | 0.1908 | No |
| 44 | RAB13 |  |  | 1416 | 0.290 | 0.1859 | No |
| 45 | HDGF |  |  | 1502 | 0.279 | 0.1704 | No |
| 46 | PLK1 |  |  | 1665 | 0.263 | 0.1327 | No |
| 47 | SMIM14 |  |  | 1854 | -0.258 | 0.0876 | No |
| 48 | RHNO1 |  |  | 2058 | -0.288 | 0.0392 | No |
| 49 | KIF11 |  |  | 2121 | -0.301 | 0.0307 | No |
| 50 | CDKN2C |  |  | 2197 | -0.313 | 0.0190 | No |
| 51 | UBALD2 |  |  | 2209 | -0.316 | 0.0252 | No |
| 52 | ANP32A |  |  | 2258 | -0.325 | 0.0214 | No |
| 53 | ARPP19 |  |  | 2261 | -0.325 | 0.0304 | No |
| 54 | ZNF106 |  |  | 2266 | -0.326 | 0.0389 | No |
| 55 | METTL7A |  |  | 2313 | -0.335 | 0.0359 | No |
| 56 | COX20 |  |  | 2369 | -0.348 | 0.0308 | No |
| 57 | RBMX |  |  | 2387 | -0.351 | 0.0364 | No |
| 58 | HSPA13 |  |  | 2446 | -0.366 | 0.0309 | No |
| 59 | IDH1 |  |  | 2526 | -0.384 | 0.0201 | No |
| 60 | MAZ |  |  | 2528 | -0.384 | 0.0311 | No |
| 61 | CDKN2D |  |  | 2548 | -0.387 | 0.0372 | No |
| 62 | NDE1 |  |  | 2588 | -0.397 | 0.0380 | No |
| 63 | MAP4K4 |  |  | 2630 | -0.408 | 0.0386 | No |
| 64 | LBR |  |  | 3068 | -0.572 | -0.0670 | No |
| 65 | MEX3A |  |  | 3069 | -0.572 | -0.0502 | No |
| 66 | DCP2 |  |  | 3100 | -0.585 | -0.0413 | No |
| 67 | HP1BP3 |  |  | 3139 | -0.612 | -0.0339 | No |
| 68 | SUGP2 |  |  | 3150 | -0.618 | -0.0184 | No |
| 69 | GAS2L3 |  |  | 3321 | -0.790 | -0.0427 | No |
| 70 | ARL6IP1 |  |  | 3339 | -0.804 | -0.0238 | No |
| 71 | PDGFRA |  |  | 3636 | -3.635 | 0.0006 | No |
Table: GSEA details [plain text format]

  

Fig 2: DOROSZKO\_U3180MG\_CLUSTER\_4: Random ES distribution      
 Gene set null distribution of ES for **DOROSZKO\_U3180MG\_CLUSTER\_4**
